# “Belly Only Pregnancy” content on social media and in internet blogs: a qualitative analysis on its definition and potential risks and benefits

**DOI:** 10.1007/s40519-022-01381-y

**Published:** 2022-03-03

**Authors:** Felizia Steube, Bernd Löwe, Angelika Weigel

**Affiliations:** Department of Psychosomatic Medicine and Psychotherapy, University Medical Center Hamburg-Eppendorf, Martinistr. 52, 20246 Hamburg, Germany

**Keywords:** Pregnancy, Social media, Body image, Eating disorders, Health information

## Abstract

**Purpose:**

Social media enlarge the impact of health and fitness trends on body image and lifestyle choices, also in birthing parents. A new and yet to investigate social media trend addressing expectant mothers is “Belly Only Pregnancy”. This qualitative study sought to define this new trend and clarify whether content related to this trend might disrupt body image or eating habits in expectant mothers.

**Methods:**

Picture and text data were gathered on a key day by screening Instagram and blog posts including or linking #bellyonlypregnancy. The identified data were categorized applying qualitative content analysis using MAXQDA software version 2018.

**Results:**

Three hundred and fifty-one Instagram and eight blog posts were included. Our qualitative analysis’ results indicated that the term “Belly Only Pregnancy” was used for describing: (1) The phenotype of an athletic woman whose abdominal size enlarges during pregnancy while not gaining excessive fat tissue. (2) An active lifestyle during pregnancy consisting of healthy nutrition and regular exercise pursuing goals like fast weight loss post-partum. Also, bodily, and mental gestational changes and the feasibility of this lifestyle were discussed.

**Conclusion:**

A “Belly Only Pregnancy” allegorizes an ideal body type for expecting mothers. Especially women with increased vulnerability for an eating disorder might be negatively affected by the consumption of content linked to this trend. However, the positive effects of a healthy diet and exercise should not be denied keeping into account the increasing prevalence of obesity and gestational diabetes.

**Level of evidence:**

Level III: Evidence obtained from cohort or case-control analytic studies.

## Introduction

Pregnancy is an exceptional situation for the female body. Structural, hormonal, and psychosocial changes cause alterations in maternal appearance, well-being, and mood [1]. For birthing parents, social media are a relevant source to meet pregnancy-related needs and find social and emotional support [2]. At the same time, pregnant women are exposed to potentially harmful pregnancy trends on social media and blogs during this period of significant alterations in body image and body (dis)satisfaction.

Through use of make-up, lighting, camera equipment, and editing programs, visual pregnancy content was found to be more staged and idealized on Instagram than other online platforms [3]. Thereby, this social media platform transports and solidifies Western ideals of beauty [4]. In non-pregnant females, previous evidence suggests that the frequency of exposure to image material on social media conforming to Western beauty ideals increases the likelihood to develop body image concerns and body dissatisfaction [5, 6]. In particular, body dissatisfaction is one of the most important risk factors for disordered eating behaviors [7]. One of the mechanisms behind the emergence of body dissatisfaction through frequent image-based social media use is the internalization of Western body ideals through social comparison [8]. With the ability to tag and link on social media postings, peer groups are tightly connected. Upward comparisons to peers that are perceived as more attractive are likely to negatively influence views of oneself [9]. For example, health and fitness content on social media is often promoted by using #fitspiration and this hashtag is becoming an increasingly popular source for young people to engage in exercise and nutrition [3, 10]. The use of diet and exercise guidelines to achieve a slim and toned body shape is seen as a predictor of discipline. Within the peer group, influencers use this comparison effect for marketing purposes by linking the achievement of the desired ideals to presented products [11]. A new visual content that addresses expectant mothers is spread under the hashtag “Belly Only Pregnancy” (BOP). This term implies a “pregnancy body ideal” for expectant mothers. The BOP trend and its potential influence on female body image during pregnancy has so far, neither been defined nor investigated.

Body image and the potential for body dissatisfaction change during the course of pregnancy. Especially the first trimester and the post-partum period are vulnerable times for a worsening body image due to their optically ambiguous transitional stage [12]. Four months post-partum, about 70% of women are attempting to lose weight because of body dissatisfaction [13]. Further, pregnancy-related weight gain due to an increase of fatty tissue (e.g., on thighs, arms, face) does not comply with common beauty ideals [14]. At the same time, pregnancy-related weight gain might even be positively evaluated and contribute to body confidence when it can be attributed to a “pregnancy beauty ideal” with a growing fetus indicated by a big belly [13] or growth of breast tissue [15]. However, in pregnant women, body dissatisfaction can lead to an over-prioritizing of appearance, the urge to breastfeed, lifestyle changes like eating in a caloric deficit to lose weight faster post-partum [4], and depression [16]. One out of 20 women is at risk for developing an eating disorder during pregnancy [17]. In this group, binge eating, anxiety, and depression are the most common symptoms [17]. To date, nature, popularity, and possible risk factors of beauty ideals on social media in the peer group of birthing parents are not yet specified. Research on this topic is necessary as social media trends can adverse dietary and exercise behaviors which might harm maternal and fetal health [4, 16, 18, 19].

From a medical point of view, the average amount of weight gain in pregnant women with a normal BMI is 9–12 kg which is accounted by about 3,5 kg of fetal weight, 1 kg of amniotic fluid, 0,5 kg of placenta weight, 1 kg of uterus enlargement, about 1,2 kg of additive maternal blood volume, 0,8 kg of breast tissue and around 4 kg of edema and fat tissue [1]. The amount of weight gain recommended during pregnancy and the associated increase in caloric intake is dependent on pre-pregnancy BMI [20]. Exercise during pregnancy lowers cesarean section rates, prevents excessive fetal and maternal gestational weight gain, and makes gestational diabetes more manageable [21]. Despite these positive effects, various studies indicated that women tend to lower their physical activity levels during pregnancy [22–24]. In non-high-risk pregnancies, moderate to high-intensity training for 30 min on most days of the week is beneficial for maternal and fetal health if adjusted to pre-pregnancy fitness levels [21].

Against this background, our investigation of “Belly Only Pregnancy” content aimed to define and shed light on this social media trend. This would provide an important first step to assess its influence on the body image of expectant mothers. Therefore, we sought to describe image and text content within the “Belly Only Pregnancy” community and examine commonalities between identified representations and suggested behaviors with evidence-based risk factors for eating disorders.

## Methods

### Data collection and processing

**Fig. 1:**
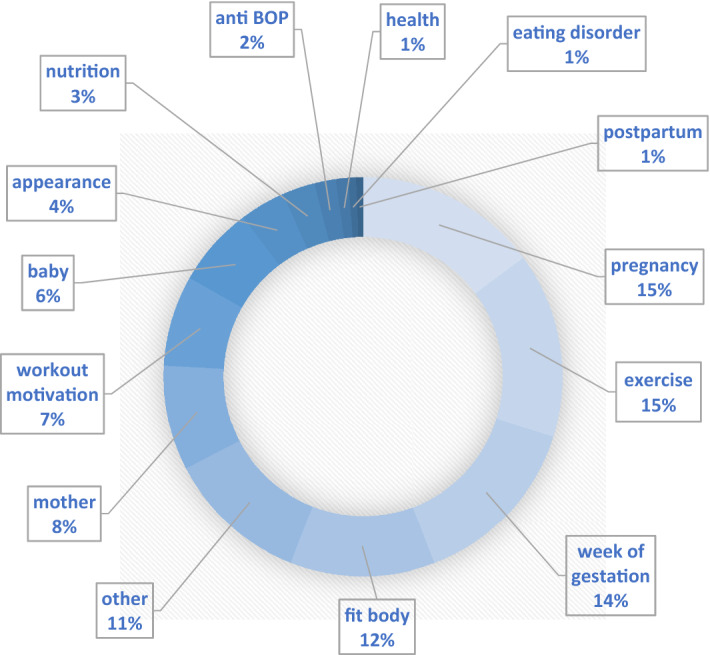
Topics of hashtags linked to #bellyonlypregnancy. Note. *BOP *Belly Only Pregnancy; hashtags linked to #bellyonlypregnancy were qualitatively summarized into the categories above according to their frequency of use

Given the fact that on Instagram body-focused social media trends have a wide range (e.g., #fitspiration [3]) and “google.de” is used to quickly get information about topics, we chose these two platforms to be the source of our data. We included all Instagram posts identified using #bellyonlypregnancy on October 24th, 2020, into the analysis. To understand the contents’ background, further information about the content creators was captured for the analysis (see Table 1). All additional hashtags to each identified post were coded and listed according to frequency in Fig. 1.

**Table 1:** General information about the included Instagram accounts

Number of included Instagram posts	351	
Number of accounts included into the analysis	124	
Posts using #bop on individual Instagram feed	1–35	Median: 1
Posts against BOP	4	
Languages of the posts	English, Polish, German, Czech, Chinese, Japanese, Korean, Spanish, Portuguese	93.85% English
Range of followers	15–95,700	Median: 451
Range of accounts subscribed to	0–5778	Median: 655
Range of posts	25–5039	Median: 557

Note *BOP *Belly Only Pregnancy

On 31st October 2020 the internet browser “google.de” was accessed to indicate websites relating to the keyword “Belly Only Pregnancy”. No filters were applied to the search. As “google.de” defines websites linked on the first results page to be the most relevant findings and assuming that users interested in “Belly Only Pregnancy” would narrow down their search given the vast amount of content available on the internet, we included those eight websites from the first page. Equivalent to the Instagram posts, these blog posts and the information about the posts’ authors were captured. Assuming that users would not only read one blog post from the website, we also included information from further blog posts from each identified blog (Table 2) which were partly directly linked on the original blogpost or proposed as an “article you might like”.

**Table 2:** General information about eight included internet blogs

Number of blogs included	8
Author’s occupations	Author, personal trainer, pre- and postnatal specialist, fitness expert, midwife, blogger, nursery schoolteacher
Language	English
Number of blogs with product recommendations	8
Number of blogs offering paid E-Books (19$-179£)	6
Blogpost against BOP	1
Number of blogs linking to Instagram	6

Note *BOP *Belly Only Pregnancy

### Qualitative approach

Text and image analysis can determine the risk potential of posts and comments on social media. In turn, this can detect eating disorder-promoting online communities [25]. Mayring postulates that qualitative content analysis can handle large amounts of material but remains qualitative-interpretative in the first step [26]. It captures the latent meaning of a subject [26] and grafts its holistic picture while defining its complexity. Because this study aims to define the trend “Belly Only Pregnancy” by looking at many different kinds of complex data, qualitative content analysis is an appropriate method. For detailed characterization of all the facets, the “Belly Only Pregnancy” movement contains, the qualitative analysis was planned to be descriptive.

### Data analysis

Screenshots of selected Instagram posts along with captions (description, subtitle) and additional hashtags as well as text data and images of the included blog posts were copied into MAXQDA Version 18 (VERBI GmbH). Image and text material was co-coded by two female researchers (AW: Ph.D., psychotherapist with prior experience and training in qualitative research, FS: medical Ph.D. student without prior experience in qualitative research). Codes were generated inductively always relating to the original material to describe the content’s general nature and to answer our research questions. A color system was created to distinguish codes created based on Instagram and/or blog content. Every hashtag was included in the analysis to be used as a quantitative synopsis. To create a cohesive code network and to narrow down the amounts of codes, categories were formed, and similar codes were summarized to extract major and minor topics. Quotations were used to illustrate the findings and identified by source (Instagram vs. blog). Major topics were mostly consistent in our findings, diverse cases in minor topics were discussed with the third author (BL) until consensus was reached.

## Results

Our search identified 351 Instagram posts using #bellyonlypregnancy published by 124 different accounts. Many influencers provided information about their occupation (79%, *n* = 98), family (52%, *n* = 65), localization (21%, *n* = 26) and a link to a website or a separate Instagram account (62%, *n* = 78). Common occupations, if indicated, were “mom” (50%, *n *= 62), “wife” (23%, *n* = 29) and terms that could be subsumed under “pregnancy/fitness expert” (5%, *n* = 6), “exercise trainer/teacher” (12%, *n* = 15), and “nurse/medical profession” (13%, *n* = 16). Some of those who mentioned a profession emphasized affiliation to the military (4%, *n* = 4). If indicated, influencers ‘countries predominantly belonged to the Western cultural sphere.

#Bellyonlypregnancy was not used as a standalone hashtag. Influencers most frequently combined it with hashtags related to fitness (e.g., #fitpregnancy, 65% of influencers). Pregnancy and baby-related hashtags such as #pregnancy, #matrescence, #babygirl, or #babyboy were also common. Hashtags we subsumed under “exercise” were utilized to express benefits of physical activity, feelings about working out, and describe the form of training (e.g., cardio vs. strength training) or exercise frequency. The week of pregnancy was also very commonly linked to #bellyonlypregnancy. Terms describing physical appearances like #babybump, #fitspo, or #absduringpregnancy and motivational hashtags such as #stayingmotivated, #goals or #alwayskeepfighting could be coded frequently. A minority used hashtags indicating an aversion to specific ideals and practices like #allbodypregnancy, #ditchdietculture, or #dietfreepregnancy. A small group combined #bellyonlypregnancy with terms referring to endocrine-, body image- or eating disorders like #hypothalamicamenhorrhea, #bodyimagewarrior, #edrecoverywarrior, or #disorderedeatingrecovery.

Accessing “google.de” on 31st October 2020, our search using the keyword “Belly Only Pregnancy” resulted in 78 300 000 hits. The first google result page comprised a total of eight internet blogs which were included in our qualitative analysis. Like our findings on Instagram, if indicated, blog authors lived in the sphere of influence of Western culture (see Table 2).

### Qualitative results

**Fig. 2:**
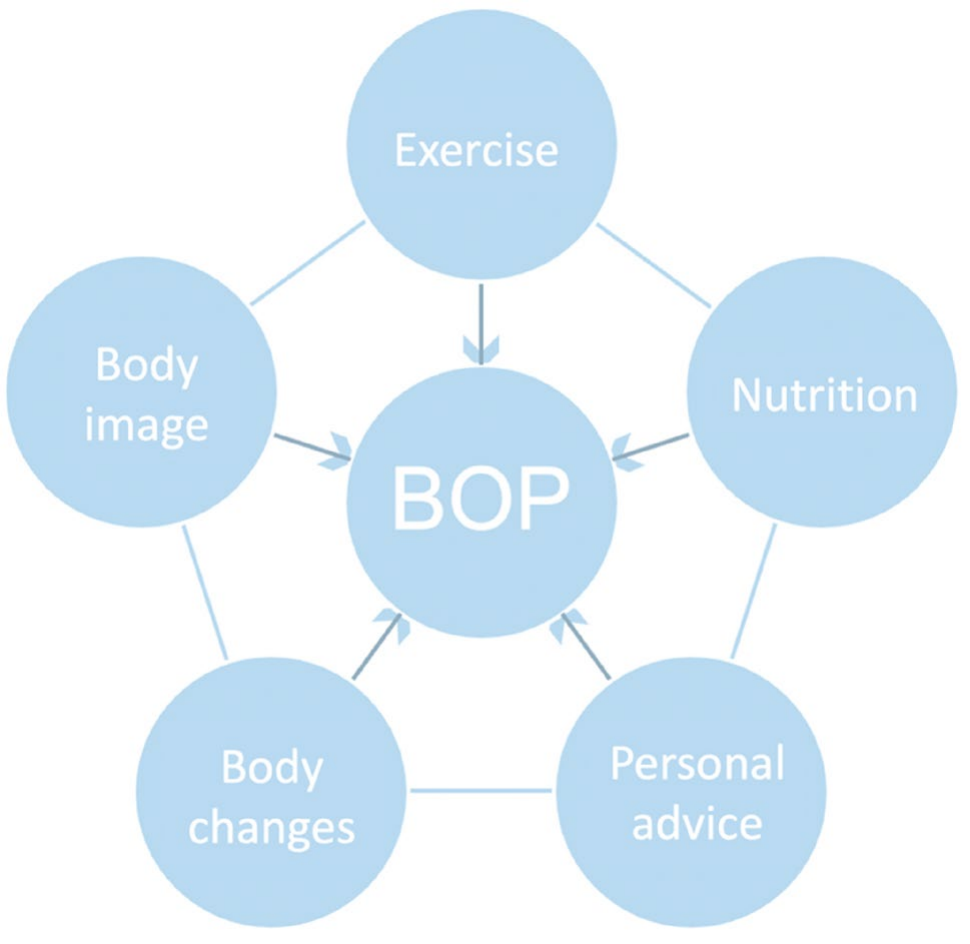
Major topics characterizing “Belly Only Pregnancy” content on Instagram and blogs Note. *BOP* Belly Only Pregnancy

Our final code network consisted of six major topics (see Fig. 2) including a detailed network of 46 subcodes and various subordinate codes aiding to define the BOP trend. Results will be presented according to the identified major topics. Anchor citations were chosen for the categories, edited for legibility, and identified by source (Instagram (I) vs. blog post (B)).

### Bodily changes during pregnancy and post-partum

Physical pregnancy changes such as nausea, strangury, muscle and backaches, exhaustion, increasing breast size, appetite irregularities, and emotional changes were common subjects of discussion. Weight gain during pregnancy was generally seen as inevitable (“It is very easy to gain weight because your hormones are playing some funny games with you. The textbook recommendation is anywhere from 25lbs-35lbs […].”; B). Contributors to increasing body weight such as growing placenta, breast tissue, uterus size, edema, or blood volume to describe changes in body shape and weight were listed. Minimizing weight gain to the lower limit of recommended value was expressed as a goal aiding to prevent birth complications, support maternal and fetal health (“[…] *a very specific diet plan that is actually super simple, yet key to gaining minimal weight while pregnant*.”; B) and to reach pre-pregnancy weight sooner after birth. A large percentage of pictures portrayed smiling women wearing a lot of make-up and tight clothing. Arms, lower back, glutes, and thighs seemed to be important body parts to keep fit and toned. Specific regimens to achieve this body shape were explained. Cellulite, stretch marks, edema, and exhaustion were rarely portrayed. Practices like measuring or usage of scales were advised to track body weight. Numbers of pregnancy weight gain and post-partum weight loss were openly shared and compared to recommended amounts and previous pregnancies (“*The most important goal is to gain a healthy amount of weight […] and keep your body trim and fit”; “So far I have gained 4.5 kg. I think that’s fine.*”; I). Postpartum medical advice addressing pelvic floor or diastasis recti rehabilitation was also addressed.

### Pregnancy and post-partum body image

**Fig. 3:**
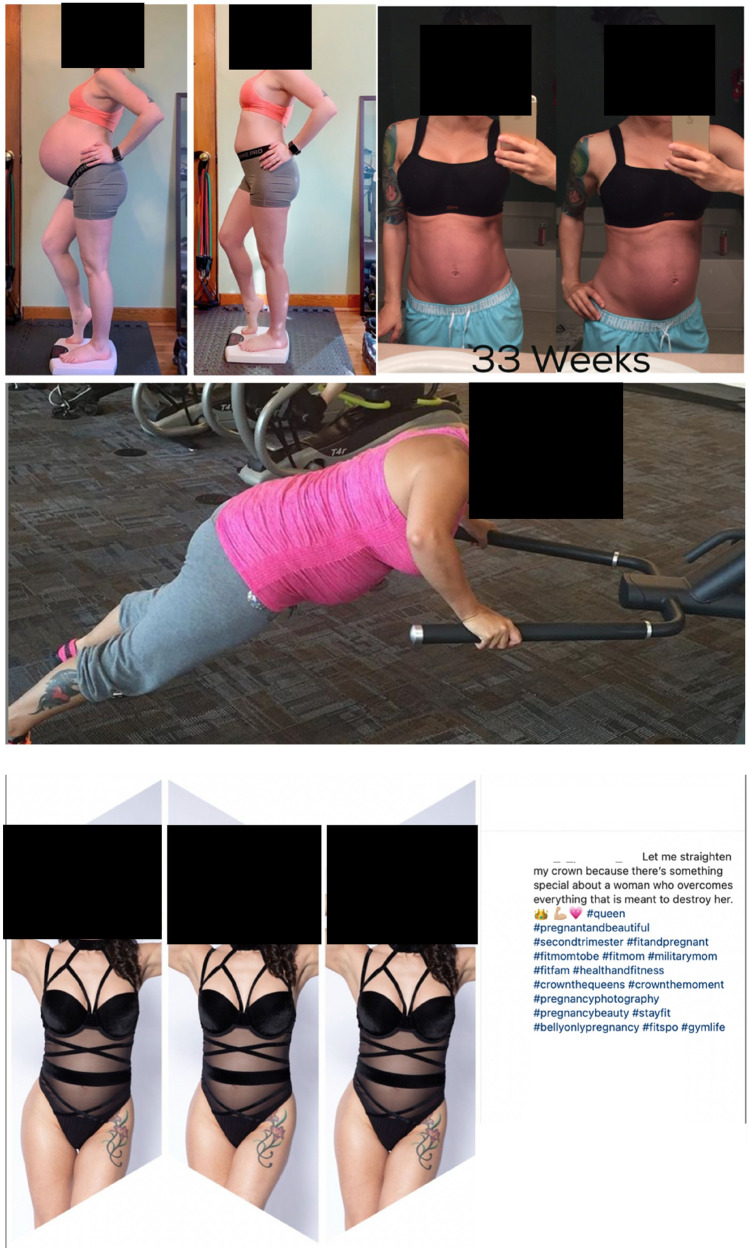
Examples of representative visual “Belly Only Pregnancy” content on Instagram and blogs

Staying fit and toned was a main matter of interest within the BOP movement making potential muscle loss or weight gain fearful for some influencers (“*I can’t run, so leg muscle has definitely faded too, and I am worried about the difficulty, starting back up when I am cleared to run again.”*; I). Defined abdominal muscles were sought after and a phenomenon observable in various images (Fig. 3). Many pregnant women pointed out that they were aiming to get their pre-pregnancy bodies and fitness levels back (“*I’ve really been watching what I eat and developing my routine again*.”; I). Images showing pregnant women from behind or straight from the front created the illusion of no belly. The desire not to look pregnant was frequently expressed (“*No one believes me when I tell them I’m almost 4 months pregnant […]”*; I). Women enjoyed getting compliments for having a BOP and were to maintain an “unpregnant “ silhouette with only a big pregnancy belly.

A small group of women reported dissatisfaction because of a changing figure and the pressure to lose pregnancy weight fast post-partum. Some of these also had a negative body image because of bullying in the past and eating disorder backgrounds (“*Growing up I got bullied for a lot of things and fat was one of them. I struggled and I still do […] to accept myself and my body for what it is*”; I).

Content creators who had already given birth proudly presented their post-partum loss of belly size often wearing underwear or tight workout attire. Women who experienced less weight gain during pregnancy described their bodies more positively than those who did not (“*But this shows that staying active throughout pregnancy is the real deal because It. Paid. Off.”*; I, see Fig. 3, picture one).

We found contents indicating a small yet present movement criticizing the BOP trend. A few pregnant women pointed out that pregnancy shifted their priorities of the body having to look a certain way to functionality and admiration for the ability to create new life. Some motivated readers to stop self-devaluation because of pregnancy changes and questioned practices like tracking calories or intense workouts. Interindividual differences during pregnancies and therefore the absurdity of comparisons was highlighted (*“All of our bodies are so different so our weight gain during pregnancy will be different as well.”*; I). The feasibility of having a BOP in the sense of gaining only belly volume and keeping the rest of the body toned and skinny was heavily called into question. Society was seen as a negative influence on body image (*“Please internet ads, stop telling me “belly only” is the golden standard of achievement when I’m growing a human. Let me take up space. I deserve it.”*; I). Key messages of material criticizing BOP were maintaining a healthy balance between exercise, eating nutritious food, and intuitive behavior while not judging the body by its appearance.

### Personal experience and advice

A positive relationship between reader and influencer was created by directly approaching readers sharing personal experiences, responding to questions, and giving pregnancy, birth, and post-partum advice (*“In order to keep your weight in your belly and even all over, you will need to work your entire body with various moves. I will share with you below my favorite moves that helped me during pregnancy.”*; B). Motivation and inspiration for specific practices such as working out, eating healthily but also accepting and valuing the female body were central elements across written data.

Especially in the first trimester, where nausea is quite common, influencers advised to listen to personal preferences and not to focus on nutrition and exercise excessively. The general recommendation was still a healthy diet and regular activity despite physical discomfort. Influencers frequently warned against the assumption that one must eat for two during pregnancy leading to excessive weight gain.

Predominantly on Instagram, women supported each other regarding bodily changes, birthing advice, and childcare. The excitement about the newborn was shared in form of baby shower and gender reveal pictures, nursery decoration, short texts, and quotes blessing the baby (*“God has blessed me tremendously with this little angel.”*; I). Despite the described anticipation of the newborn, the analyzed material focused less on the relationship between parents and child but more on the mother’s appearance and lifestyle.

Many influencers, especially on blogs, used their range of influence to earn money with product placements. Pregnancy and specific health and fitness e-books, maternity wear, belly bandages, snacks, and protein powders were recommended using the BOP trend for marketing purposes.

### Exercise

A common tip for having a BOP was to work out throughout the whole pregnancy. Exercise recommendations could be divided into cardio training such as swimming or walking and strength training like Pilates or weightlifting. The average recommended exercise frequency was five times a week for about thirty minutes.

Visual content demonstrating strength training focused on lower body parts and arms, but abdominal and core workouts titled “pregnancy safe” were also available. A vast majority of influencers incorporated walking a certain number of steps (usually 8–10 k) a day. Benefits of exercise during pregnancy like being strong for safe delivery, increasing maternal and fetal health, strengthening pelvic floor muscles, preventing diastasis recti, and faster recovery post-partum were explained. Several influencers shared activity levels in form of diaries or screenshots of tracking watch displays showing daily steps, numbers of lifted weights during a workout, and burned calories.

On Instagram, one woman kept working out despite having cramps in the abdominal area, some expressed feelings of guilt for not sticking to a workout schedule due to the fear of gaining weight or potential muscle loss. Working out was seen as an important factor or even as a precondition for having a safe delivery. Text material emphasized that pregnancy was a reason to start an active lifestyle (*“A baby isn’t an excuse to be lazy it’s a reason to get up and move.*”; I). Mainly on blogs, it was highlighted that exercise during pregnancy should be agreed with a doctor.

### Nutrition

Many pregnant women pointed out that the possibility to “Eat for two” during pregnancy was an outdated assumption. Influencers educated readers about increasing their caloric intake only starting in the second trimester for up to 25%. Examples of well-balanced food ideas were presented to reach macronutrients. To prevent overeating, it was advised to eat five to six small meals a day and to stay hydrated. Overly sweet, fattening, and fast foods should be avoided. Calorie numbers of snack and meal ideas were indicated (*“Know your calories! This is the most important tip of all you have got to get a grip on how many calories you should be consuming per day. […] Use this calculator to help determine your needs”*; B). Bloggers stressed that tracking calories to control weight gain was a necessity (“*I know it’s hard to lose weight so better control it before it is too late.”*; B). It was implicated that sleep quality increased through a balanced diet and exercise improving overall well-being, relaxing the child, preventing binging on food.

Blog authors highlighted that their nutritional tips were based on personal experience and therefore advised to consult registered dieticians if needed.

### Minor topics

A minority of influencers used #bellyonlypregnancy even though they were visibly overweight. Some accentuated regret for not having a BOP or struggled because of more weight gain compared to those having bodies according to the BOP trend.

Black and indigenous people of color as content creators were rarely represented in our data.                            Furthermore, wedding rings were often portrayed while partners were rarely seen.

On Instagram, a small proportion of influencers using #bellyonlypregnancy posted images of baby shower gifts, baby clothing, nursery rooms, streets, or nature.

## Discussion

The “Belly Only Pregnancy” trend portrays the image of a fit pregnant woman who consistently controls her diet and physical activity to achieve the lowest possible weight gain during and rapid weight loss after pregnancy maintaining a muscular physique. The dietary, exercise and personal advice marketed by influencers include suggestions for lifestyle changes to achieve this “pregnancy beauty ideal”. However, this is unattainable for many pregnant women. This can change the body image of those pregnant women exposed to BOP content.

In our data, especially women describing body image issues, excessive gestational weight gain, or bullying in the past seemed to experience body dissatisfaction, a well-established risk factor for the development of disordered eating behaviors [27]. This was reflected in our findings through regular determination of body measurements, counting calories, and maintaining or increasing (excessive) physical activity during pregnancy and after delivery. The fear of gaining and/or not being able to lose weight quickly post-partum was a major concern. These behaviors are in line with the theoretical construct of body dissatisfaction that assumes that the exposure to unrealistic and often sexualized beauty ideals in social networks leads to self-objectification, appearance comparisons, internalization, and body monitoring [8]. This suggests that expectant mothers—particularly those with an increased vulnerability for an eating disorder—might perceive body dissatisfaction after the consumption of content related to the BOP trend. Future studies should further investigate this finding.

Most BOP influencers whose content we analyzed did not actively promote restrictive eating habits. However, BOP diets, consisting of strict meal plans and daily calorie restrictions, did not seem to be intuitive. In women with an eating disorder history, these contents could trigger a relapse, especially when fearing weight gain and strongly urging to control exercise and food intake [28, 29]. These lifestyle choices can have harmful consequences. Disordered eating behavior like malnutrition, especially micronutrient-, vitamin- and caloric restriction during pregnancy can have fatal consequences for the course of pregnancy and fetal outcome [18].

The majority of the sports and training intensities to pursue a BOP lifestyle found in our data were in line with current guidelines when compared to physical activity recommendations during pregnancy [30]. Some women pursued questionable exercise routines like abdominal and core workouts and long walks despite experiencing pain. Even though many women followed a very strict fitness schedule and the pregnancy-related reduction in exercise intensity was a stress factor for some, our data analysis is not conclusive enough to judge whether the sporting behavior was compulsive. However, the number of women who seemed to define themselves strongly by their physical activity was very large. Therefore, women should be educated about short-term effects such as fetal bradycardia and the possible risks of extreme exercise [19]. Especially females who experienced body dissatisfaction or disordered eating in the past could be vulnerable to relapsing given that high exercise levels, and visible physique and skill progress, were proudly shared within the BOP movement.

In addition to the potential dangers of lifestyle changes marketed by influencers, frequent social media use itself, especially active photo uploading, negative comments, and body and lifestyle comparisons [31], are associated with body monitoring, thin-ideal internalization, and self-objectification possibly leading to body image issues and eating disorders [32, 33]. Similarly, regular exposure to BOP content, depicting athletic pregnant women with rapid post-partum weight loss, might create pressure among the peer group of birthing parents to achieve this beauty ideal through lifestyle changes like increasing exercise levels and monitoring food intake. In previous studies on social media exposure in non-pregnant young women, a positive association was found between upward comparisons and body monitoring with disordered eating behavior [34]. Further, exposure to social media trends leads to negative moods, sadness, and feelings of guilt resulting in body dissatisfaction [35]. This is particularly important in relation to one target group of our findings, as post-partum women are generally more vulnerable to the development of body dissatisfaction [3].

Despite the references that could be made between the material studied and evidence-based risk factors for the development of eating disorders, (i.e., thin-ideal internalization and body dissatisfaction) it should be considered that the numbers of women suffering from diet-induced gestational diabetes and the consequences of excessive gestational weight gain are rising [36]. Within the BOP community, the “Eating for two” mentality was strongly rejected. Calorie requirements during pregnancy increase only by a small amount [37]. Therefore, calling for a conscious calorie intake could lower the rates of excessive gestational weight gain and gestational diabetes. Further, regular exercise during pregnancy lowers musculoskeletal complaints, the risk of depression, caesarian section rates [38] and reduces the risk of hypertensive pregnancy diseases [30]. Regular exercise also improves cardiovascular and lung function as well as sleep quality [30]. Moreover, digital education and supportive exchanges among women on social media about nutrition and exercise have a positive impact on the prevention of excessive gestational weight gain [39], increase activity levels [40], lower depressive symptoms [41], and contributes to rising maternal and fetal health levels [42].

Despite the possible advantages of a balanced diet and an active lifestyle especially for expectant mothers, the harmful potential for thin-idealization, body dissatisfaction, and possibly the development of an eating disorder are dangerous aspects of the BOP trend. In addition to the negative effects on eating behaviour, the potential influence of this trend on (pre-existing) comorbidities such as affective and anxiety disorders should also be considered [43]. Regardless of how positively influencers implement this trend, some vulnerable peers might harm themselves and the unborn child by interacting with the BOP content.

### Strength and limits

Data for this work were collected on two key dates on Instagram and via google.de and new content is posted on social media every day. However, we coded a vast amount of data uploaded in different years and produced from many different influencers. Thus, our key date approach identified relevant material posted in the last years. Further, we included the image-based social media platform Instagram, which carries a high risk for thin-ideal internalization [44] and developing an eating disorder [45], and blogs that are run by the most popular creators of pregnancy workout and nutrition e-guides undermining the relevance of our studies’ results. Due to the present study’s focus on a descriptive picture of BOP, we could only hypothesize on the actual impact of BOP content on pregnant women, and further research is urgently needed to empirically investigate the hypothesizes.

## What is already known on this subject?

To our best knowledge, there is currently no research on the topic of BOP that could thematically embed our study. This study was necessary to understand health and fitness social media trends with the target population of pregnant women and to create awareness of possible benefits and risks. Future studies are essential to investigate the effects of the BOP trend on pregnant women, for example, the unintentional consumption of BOP content and short-term relations between BOP exposure and changes in body (dis)satisfaction (e.g., with the use of ecological momentary assessments). These insights could inform the development of preventive interventions as well as health literacy for pregnant women.

## What does our study add?

A “Belly Only Pregnancy” means maintaining a defined body through exercise and nutrition regimes during pregnancy striving to ensure a complication-free birth and regain a slim and toned appearance as soon as possible post-partum. Exercise and nutrition during pregnancy as the trend’s central elements illustrate the strong focus on appearance which in such a vulnerable period of bodily and mental changes was discussed controversially in some of our material. According to current guidelines, pregnant women are advised to be physically active regularly [21] and to eat a balanced diet [46]. This is particularly important and correct given the rising rates of gestational diabetes and obesity worldwide. At the same time, pregnancy health and fitness trends such as BOP are becoming popular on social media, putting (unconscious) pressure on (expectant) mothers to achieve or maintain a trained body through strict exercise and dietary recommendations. Especially women who already have body image issues may be vulnerable to developing an eating disorder through exposure to content associated with the BOP trend.

The following clinical implications can be derived from our work:

- There is a great need to sensitize practitioners (gynecologists, maternity nurses, other health professionals working with pregnant women) to potentially body image-altering risks of social media consumption in the peer group of pregnant women. Practitioners need to point out possible harmful influences on maternal and fetal health, which to date receive little attention in everyday clinical practice.
- In the longer term, (digital) education material should be developed to provide information about the risks associated with the consumption of BOP and social media content during pregnancy.

## Data Availability

The dataset generated and analyzed during the current study is available from the corresponding author on reasonable request.

## References

[1] Weyerstahl T, Stauber M, Goerke K, Steller J, Valet A (2013). Klinikleitfaden Gynäkologie, Geburtshilfe. Duale Reihe Gynäkologie.

[2] Haslam DM, Tee A, Baker S (2017). The use of social media as a mechanism of social support in parents. J Child Fam Stud.

[3] Boepple L, Thompson JK (2016). A content analytic comparison of fitspiration and thinspiration websites. Int J Eat Disord.

[4] Fuller-Tyszkiewicz M, Skouteris H, Watson BE, Hill B (2013). Body dissatisfaction during pregnancy: a systematic review of cross-sectional and prospective correlates. J Health Psychol.

[5] Haferkamp N, Krämer NC (2011). Social comparison 2.0: examining the effects of online profiles on social-networking sites. Cyberpsychol Behav Soc Network.

[6] Bair CE, Kelly NR, Serdar KL, Mazzeo SE (2012). Does the Internet function like magazines? An exploration of image-focused media, eating pathology, and body dissatisfaction. Eat Behav.

[7] Aparicio-Martinez P, Perea-Moreno AJ, Martinez-Jimenez MP, Redel-Macías MD, Pagliari C, Vaquero-Abellan M (2019). Social media, thin-ideal, body dissatisfaction, and disordered eating attitudes: an exploratory analysis. Int J Environ Res Public Health.

[8] Holland G, Tiggemann M (2016). A systematic review of the impact of the use of social networking sites on body image and disordered eating outcomes. Body Image.

[9] Perloff RM (2014). Social media effects on young women’s body image concerns: theoretical perspectives and an agenda for research. Sex Roles.

[10] Lupton D (2021). Young people’s use of digital health technologies in the global north: narrative review. J Med Internet Res.

[11] Pilgrim K, Bohnet-Joschko S (2019). Selling health and happiness how influencers communicate on Instagram about dieting and exercise: mixed methods research. BMC Public Health.

[12] Hodgkinson EL, Smith DM, Wittkowski A (2014). Women’s experiences of their pregnancy and postpartum body image: a systematic review and meta-synthesis. BMC Pregnancy Childbirth.

[13] Zerwas SC, Claydon EA |(2014) Women’s Reproductive Mental Health across the Lifespan. Springer International Publishing AG; Cham, Switzerland. Eating Disorders Across the Life-Span: From Menstruation to Menopause; pp. 237–261

[14] Nash M (2012). Weighty matters negotiating ‘fatness’ and ‘in-betweenness’ in early pregnancy. Femin Psychol.

[15] Chang SR, Chao YM, Kenney NJ (2006). I am a woman and i’m pregnant: body image of women in Taiwan during the third trimester of pregnancy. Birth.

[16] Clark A, Skouteris H, Wertheim EH, Paxton SJ, Milgrom J (2009). The relationship between depression and body dissatisfaction across pregnancy and the postpartum: a prospective study. J Health Psychol.

[17] Martínez-Olcina M, Rubio-Arias JA, Reche-García C, Leyva-Vela B, Hernández-García M, Hernández-Morante JJ, Martínez-Rodríguez A (2020). Eating disorders in pregnant and breastfeeding women: a systematic review. Medicina.

[18] Pitkin RM (1977). Nutritional influences during pregnancy. Med Clin North Am.

[19] Salvesen KÅ, Hem E, Sundgot-Borgen J (2012). Fetal wellbeing may be compromised during strenuous exercise among pregnant elite athletes. Br J Sports Med.

[20] Rasmussen KM, Yaktine AL, Institute of Medicine (US) and National Research Council (US) Committee to Reexamine IOM Pregnancy Weight Guidelines (Eds.). (2009). Weight Gain During Pregnancy: Reexamining the Guidelines. National Academies Press (US) 20669500

[21] Hinman SK, Smith KB, Quillen DM, Smith MS (2015). Exercise in pregnancy: a clinical review. Sports Health.

[22] Evenson KR, Wen F (2011). Prevalence and correlates of objectively measured physical activity and sedentary behavior among US pregnant women. Prev Med.

[23] Hegaard HK, Damm P, Hedegaard M, Henriksen TB, Ottesen B, Dykes AK, Kjaergaard H (2011). Sports and leisure time physical activity during pregnancy in nulliparous women. Matern Child Health J.

[24] Amezcua-Prieto C, Olmedo-Requena R, Jímenez-Mejías E, Hurtado-Sánchez F, Mozas-Moreno J, Lardelli-Claret P, Jiménez-Moleón JJ (2013). Changes in leisure time physical activity during pregnancy compared to the prior year. Matern Child Health J.

[25] Moessner M, Feldhege J, Wolf M, Bauer S (2018). Analyzing big data in social media: Text and network analyses of an eating disorder forum. Int J Eat Disord.

[26] Mayring P, Fenzl T (2019). Qualitative Inhaltsanalyse. Handbuch Methoden Der Empirischen Sozialforschung.

[27] McLean SA, Paxton SJ (2019). Body image in the context of eating disorders. Psychiatr Clin North Am.

[28] Berends T, Boonstra N, van Elburg A (2018). Relapse in anorexia nervosa: a systematic review and meta-analysis. Curr Opin Psychiatry.

[29] Koller KA, Thompson KA, Miller AJ, Walsh EC, Bardone-Cone AM (2020). Body appreciation and intuitive eating in eating disorder recovery. Int J Eat Disord.

[30] Oliveira C, Imakawa T, Moisés E (2017) Physical activity during pregnancy recommendations and assessment tools. Atividade física durante a gestação: recomendações e ferramentas de avaliação. Revista brasileira de ginecologia e andomizedn revista da Federacao Brasileira das Sociedades de Ginecologia e Obstetricia 39(8): 424–432. 10.1055/s-0037-1604180 10.1055/s-0037-1604180 PMC10316950 28783859

[31] Hogue JV, Mills JS (2019). The effects of active social media engagement with peers on body image in young women. Body Image.

[32] Manago AM, Ward L, Lemm KM, Reed L, Seabrook R (2015). Facebook involvement, objectified body consciousness, body shame, and sexual assertiveness in college women and men. Sex Roles.

[33] Vandenbosch L, Eggermont S (2012). Understanding sexual objectification: A comprehensive approach toward media exposure and girls’ internalization of beauty ideals, self-objectification, and body surveillance. J Commun.

[34] Saunders JF, Eaton AA (2018). Snaps, selfies, and shares: how three popular social media platforms contribute to the sociocultural model of disordered eating among young women. Cyberpsychol Behav Soc Netw.

[35] Bennett BL, Whisenhunt BL, Hudson DL, Wagner AF, Latner JD, Stefano EC, Beauchamp MT (2020). Examining the impact of social media on mood and body dissatisfaction using ecological momentary assessment. J Am Coll Health.

[36] Magro-Malosso ER, Saccone G, Di Tommaso M, Roman A, Berghella V (2017). Exercise during pregnancy and risk of gestational hypertensive disorders: a systematic review and meta-analysis. Acta Obstet Gynecol Scand.

[37] Butte NF, King JC (2005). Energy requirements during pregnancy and lactation. Public Health Nutr.

[38] Gregg VH, Ferguson JE (2017). Exercise in Pregnancy. Clin Sports Med.

[39] Willcox JC, Wilkinson SA, Lappas M, Ball K, Crawford D, McCarthy EA, Fjeldsoe B, Whittaker R, Maddison R, Campbell KJ (2017). A mobile health intervention promoting healthy gestational weight gain for women entering pregnancy at a high body mass index: the txt4two pilot randomized controlled trial. BJOG.

[40] Hayman M, Reaburn P, Browne M, Vandelanotte C, Alley S, Short CE (2017). Feasibility, acceptability and efficacy of a web-based computer-tailored physical activity intervention for pregnant women - the Fit4Two randomised controlled trial. BMC Pregnancy Childbirth.

[41] Lau Y, Htun TP, Wong SN, Tam W, Klainin-Yobas P (2017). Therapist-supported internet-based cognitive behavior therapy for stress, anxiety, and depressive symptoms among postpartum women: a systematic review and meta-analysis. J Med Internet Res.

[42] Poorman E, Gazmararian J, Parker RM, Yang B, Elon L (2015). Use of text messaging for maternal and infant health: a systematic review of the literature. Matern Child Health J.

[43] Ulfvebrand S, Birgegård A, Norring C, Högdahl L, von Hausswolff-Juhlin Y (2015). Psychiatric comorbidity in women and men with eating disorders results from a large clinical database. Psychiatry Res.

[44] Cohen R, Newton-John T, Slater A (2017). The relationship between Facebook and Instagram appearance-focused activities and body image concerns in young women. Body Image.

[45] Turner PG, Lefevre CE (2017). Instagram use is linked to increased symptoms of orthorexia nervosa. Eat Weight Disord.

[46] Lowensohn RI, Stadler DD, Naze C (2016). Current concepts of maternal nutrition. Obstet Gynecol Surv.

